# Importance of immunoglobulin therapy for COVID-19 patients with lymphocytopenia

**DOI:** 10.1186/s42269-021-00502-4

**Published:** 2021-02-22

**Authors:** Heba K. Nabih

**Affiliations:** Medical Biochemistry Department, Medical Research Division, National Research Centre, 33 El Bohouth St. (Former El Tahrir St.), Dokki, P.O. 12622, Giza, Egypt

**Keywords:** COVID-19, Lymphocytopenia, Immunoglobulins, Immunotherapy, Passive immunization, Convalescent plasma therapy

## Abstract

**Background:**

The global coronavirus disease 2019 (COVID-19) was announced as pandemic by the World Health Organization (WHO). With the increased number of infected and dead victims daily all over the world, it becomes necessary to stop or overcome its rapid spread.

Main body

Although the production of vaccine or even specified effective anti-virus may take about six months to a year, intravenous immunoglobulin (IVIg) may be clinically used as a safe treatment to save and improve the quality of life of patients with a variety of immunodeficiency diseases such as lymphocytopenia, which is a common clinical feature in COVID-19.

**Conclusion:**

Through the current review, it was concluded that this passive immunization may promote the immunity to better fight against the virus, so the survival of the patients could be kept longer. The efficacy of immunotherapy with IVIg would be greater if the immune IgG antibodies were collected from convalescent plasma therapy.

## Background

The epidemic of severe acute respiratory syndrome coronavirus 2 (SARS-CoV-2) originating in Wuhan, China, has rapidly spread all over the world (Huang et al. 2020), and in March 2020, the World Health Organization (WHO) declared coronavirus disease 2019 (COVID-19) (SARS-CoV-2) outbreak a pandemic (World Health Organization (WHO) 2020).

## Main text

### Incidence of lymphocytopenia in COVID-19 patients

One of the most common clinical features of patients suffering from novel coronavirus disease 2019 (COVID-19) is lymphocytopenia. Such a decrease of lymphocytes in many cases may indicate or lead to serious illness that can be fatal due to suppression of immunity that will result in severe risk complications due to increased facility of viral, bacterial, and fungal infections. In one screening study including 41 patients diagnosed with COVID-19, Huang et al. 2020 recorded that 26 (63%) of 41 patients had lymphocytopenia (85% of an intensive care unit (ICU) patients and 54% of non-ICU patients). Thirteen (32%) patients were admitted to ICU and six (15%) died. Compared with non-ICU patients, ICU patients had higher plasma levels of interleukins (ILs)-2, 7, 10, granulocyte–macrophage colony-stimulating factor (GM-CSF), and tumor necrosis factor alpha (TNF-α) (Huang et al. 2020).

### Immunoglobulins and their production

Immunoglobulins (Igs) are highly diverse autologous molecules able to improve the immunity in different physiological and diseased conditions. They have a role in the development and function of both B and T lymphocytes. Deficiencies in either of these two arms of immunity can result in a heightened susceptibility to bacterial, fungal, or viral infections. Immunoglobulins are a group of closely related glycoproteins composed of 82–96% protein and 4–18% carbohydrate. The plasma concentration of these 150 kDa glycoproteins is depending on individual variations and level of environmental exposure to antigens, with a mean concentration of 7 to 12 g/L. The immunoglobulin G (IgG), a major effector molecule of the humoral immune response, accounts for about 75% of the total Igs in the plasma of healthy individuals. Other classes of Igs which constitute the other 25% of the Igs are IgM, IgA, IgD, and IgE (Afonso and João 2016).

Polyclonal immunoglobulins may be used as a therapy in many diseases in different circumstances such as primary and secondary hypogammaglobulinemia, recurrent infections, polyneuropathies, cancer, and after allogeneic transplantation. Moreover, polyclonal immunoglobulins were reported to activate certain subpopulations of T cells with effects on T cell proliferation, survival, and function in lymphocytopenia, a situation that is accompanied by hypogammaglobulinemia. These results confirm the impact of intravenous immunoglobulin (IVIg) treatment as a therapy in cases of severe lymphocytopenia, a situation that can occur after chemo- and radiotherapy treatments of cancer patients with or without COVID-19 infection. Besides, IVIg has also been used as an anti-infectious agent against viruses, bacteria, and fungi in human patients and experimental models (Diep et al. 2016; Bayry et al. 2004; Shopsin et al. 2016; Ben-Nathan et al. 2003). IVIgs are a therapeutic preparation of pooled normal polyspecific human IgGs collected from large numbers of healthy donors. The preparation contains antibodies to microbial antigens, self-antigens (natural auto-antibodies), and anti-idiotypic antibodies which recognize other antibodies. The many thousands of donors who contribute to a typical pool of plasma used for isolation of immunoglobulin represent a wide range of antibody specificities against infectious agents (Looney and Huggins 2006; Guideline and on the Clinical Investigation of Human NormalImmunoglobulin For Intravenous Administration (IVIg) 2014) such as bacterial, viral, and also a large number of self-antigens reflecting the cumulative exposure of the donor population to the environment. IVIg preparations from pooled plasma of thousands of healthy donors contain both monomeric IgG fraction that shows increased reactivity toward T-independent antigens of pathogens and dimeric IgG fraction that represents mainly idiotypic–anti-idiotypic antibody pairs and controls the immune response to certain categories of pathogens, whereas IVIg isolated from one donor contains only IgG monomers (Tankersley et al. 1988). Cohn et al. in cooperation with Oncley et al. (Cohn et al. 1946; Oncley et al. 1949) found a method that yields Igs for intravenous and subcutaneous use (Eibl 2008) by the separation of plasma proteins into individual stable large-scale fractions with different biologic functions. The primary purification processes for commercial IVIg production include fractionation, purification (through removal of lipoproteins, albumin, and other plasma proteins, by cloth filtration in the presence of filter aid, a procedure that also removes many potential viruses (Buchacher and Iberer 2006)), ion-exchange chromatography (the implementation of chromatographic procedures also aims at the removal of IgA, a cause of severe anaphylactic shock in patients with IgA deficiency presenting anti-IgA antibodies), and virus inactivation and removal using solvent/detergent (S/D) treatment (Fahey and Horbett 1959). Pasteurization is used by some manufacturers to treat IVIg solutions by heating at 60 ˝C for 10 h (Uemura et al. 1994). A more recent method prepares the IgG more or less directly from plasma and not from a precipitate. Recently, the manufacturing processes have become more developed ensuring good in vivo tolerance and minimizing side effects, in particular transmission of infectious agents and facilitated recovery (Radosevich and Burnouf 2010; Martin 2006).

Depending on the immune status of the patient, the half-life of IVIg after intravenous infusion or intramuscular injection is about 2–3 weeks. The half-life of most IgG antibodies is 33–36 days in immune-deficient patients who receive IVIg infusions, with a median serum half-life of 23 days (Wasserman et al. 2009). This increases the availability of sufficient specific IgG to fight infection. After injection of relatively high amounts of IVIg (0.1–2 g/kg body mass), the IgG concentration in serum immediately elevates, falls rapidly in the first 1 to 7 days (due to passage of IgG out of the vasculature into lymph and extracellular fluid compartments), and then falls more slowly thereafter (because of catabolism while IgG in lymph and tissues slowly diffuses back into the circulation) (Bonilla 2008).

### Impact of immunoglobulin therapy for COVID-19 patients

IVIg has immunomodulatory activities such as decreasing self cellular destruction mediated by phagocytosis (Gelfand 2012), neutralizing an autoantibody and hampering its production via binding to auto-reactive B lymphocytes (Dwyer 1992), complement system inhibition to hamper the subsequent formation of the membrane attack complex (Basta 1996), modulation of cytokine including interleukins (ILs)-1, 2, 3, 4, 5, 10, tumor necrosis factor alpha (TNF-α), and granulocyte–macrophage colony-stimulating factor (GM-CSF) and cytokine antagonist (IL-1 receptor antagonist) production (Andersson et al. 1996), impairing the ability of mature dendritic cells (DCs) to produce IL-12, and enhancing their ability to produce IL-10 (Bayry et al. 2003; Othy et al. 2012), preventing platelet consumption triggered by a pathogenic auto-antibody (Samuelsson et al. 2001), enhancing in glucocorticoid receptor-binding affinity, subsequently enhanced glucocorticoid sensitivity, and synergistic suppression of lymphocyte activation when combined with glucocorticoids (Spahn et al. 1999), and expanding of lymphocyte repertoire diversity to promote lymphocyte development and also to improve lymphocyte function (Pires et al. 2010). Additionally, IVIg therapy also led to increased expression of peroxisome proliferator-activated receptor gamma (PPARγ), a ligand-activated transcription factor that mediates anti-inflammatory functions and resolution of inflammation, but reduced Toll-like receptor 4 (TLR-4) expression, which mediates the inflammatory response (Jawhara 2020). All of these IVIg activates could help in enhancing production and improving function of lymphocytes, relieving the associated inflammations, and protecting cells of patients suffering from novel COVID-19.

Less than 5 percent of patients have adverse reactions to IVIg due to the aggregation forms of IgGs (Gelfand 2012; Bayry et al. 2003). Mild reactions to Igs can occur within the first 30 min after infusion and may be relieved by minimizing the infusion rate or temporarily stopping the infusion (Carbone 2007). Moreover, a number of clinical and experimental studies show that these adverse reactions can be reduced by subcutaneous administration of IVIg (Ochs et al. 2006; Markvardsen et al. 2013; Harbo et al. 2010).

### Convalescent plasma therapy for COVID-19 patients

Immunotherapy with IVIg could be employed to neutralize COVID-19. However, the efficacy of IVIg would be better if the immune IgG antibodies were collected from patients who have recovered from COVID-19 (convalescent plasma therapy) in the same city, or the surrounding area (as these donor subjects have naturally been confronted with the virus), in order to increase the chance of neutralizing the virus (passive immunization) until stronger options such as vaccines that will may take long time to develop and test before it can be public or effective antiviral therapy against COVID-19 are available (Jawhara 2020). All donors have to be previously diagnosed with laboratory-confirmed COVID-19 and subsequently tested negative for SARS-CoV-2 and other respiratory viruses, as well as for hepatitis B virus, hepatitis C virus, HIV, and syphilis at the time of blood donation. In one case series, five patients who were critically ill with COVID-19 were treated with convalescent plasma. All plasma samples from the donors were detected to have high virus-specific IgG and IgM ELISA titers, antibodies. The results of this study lead to that convalescent plasma may have contributed to the clearance of the COVID-19 virus and also the improvement of symptoms (Shen et al. 2020).

## Conclusions

Intravenous immunoglobulin (IVIg) therapy has saved and improved the quality of life of patients with a variety of immunodeficiency diseases such as lymphocytopenia, which is a common clinical feature in COVID-19. Despite an overall excellent safety record, IVIg can cause serious complications that may be minimized. The science must keep moving so that convalescent plasma, which is isolated from recently COVID-19-recovered survivors, should be collected. The immunoglobulins must be quickly characterized and then produced efficiently in large quantities through expert scientific companies and pharmaceutical manufacturers. These immunoglobulins are safe and effective against COVID-19 and will attenuate the fatal threat and keep life of COVID-19 patients.

## Data Availability

Not applicable.

## Abbreviations

COVID-19: Coronavirus disease 2019
WHO: World Health Organization
IVIg: Intravenous immunoglobulin
ICU: Intensive care unit
ILs: Interleukins
GM-CSF: Granulocyte–macrophage colony-stimulating factor
TNF-α: Tumor necrosis factor alpha
Igs: Immunoglobulins
IgG: Immunoglobulin G
S/D: Solvent/detergent
DCs: Dendritic cells
PPARγ: Peroxisome proliferator-activated receptor gamma
TLR-4: Toll-like receptor 4
